# An Engineered Mouse Model That Generates a Diverse Repertoire of Endogenous, High-Affinity Common Light Chain Antibodies

**DOI:** 10.3390/antib13010014

**Published:** 2024-02-08

**Authors:** Yinghui Rong, I-Ling Chen, Lance Larrabee, Manali S. Sawant, Germaine Fuh, Patrick Koenig

**Affiliations:** 23andMe, Inc. Therapeutics, 349 Oyster Point Boulevard, South San Francisco, CA 94080, USA; yinghuir@23andme.com (Y.R.); lancel@23andme.com (L.L.); gfuh@23andme.com (G.F.)

**Keywords:** antibody discovery, genetically engineered mouse model, common light chain, bispecific antibodies

## Abstract

Bispecific antibodies have gained increasing popularity as therapeutics as they enable novel activities that cannot be achieved with monospecific antibodies. Some of the most popular bispecific formats are molecules in which two Fab arms with different antigen specificities are combined into one IgG-like molecule. One way to produce these bispecific molecules requires the discovery of antibodies against the two antigens of interest that share a common light chain. Here, we present the generation and characterization of a common light chain mouse model, in which the endogenous IGKJ cluster is replaced with a prearranged, modified murine IGKV10-96/IGKJ1 segment. We demonstrate that genetic modification does not impact B-cell development. Upon immunization with ovalbumin, the animals generate an antibody repertoire with VH gene segment usage of a similar diversity to wildtype mice, while the light chain diversity is restricted to antibodies derived from the prearranged IGKV10-96/IGKJ1 germline. We further show that the clonotype diversity of the common light chain immune repertoire matches the diversity of immune repertoire isolated from wildtype mice. Finally, the common light chain anti-ovalbumin antibodies have only slightly lower affinities than antibodies isolated from wildtype mice, demonstrating the suitability of these animals for antibody discovery for bispecific antibody generation.

## 1. Introduction

Bispecific antibodies have gained increased popularity as they enable novel activities that cannot be achieved with regular monospecific antibodies [1]. Various formats have been established in recent years covering a variety of binding stoichiometries and specificities [2]. Among the most popular formats are IgG-like bispecifics which resemble the structure of a regular IgG combining two Fab arms of different specificities. Numerous IgG-like bispecifics are currently in clinical development or have been approved [3].

One difficulty in generating an IgG-like bispecific is the requirement to express four different chains—two different heavy chains and two different light chains—which often can form tetramers of multiple combinations resulting in the desired tetrameric IgG-like bispecific with correctly paired heavy and light chains in a mixture with unwanted byproducts that are difficult to remove. Several antibody engineering solutions have been described, which utilize linkers [4], the exchange [5] or replacement [6,7] of the constant heavy chain domain 1 (CH1) and/or the light chain constant domain (CL) to overcome the heavy and light chain pairing problem. However, most of these formats result in bispecific antibodies which show large deviations in the primary sequence from wildtype IgG antibodies. In contrast, several approaches have been established which require no or only minor modifications to the antibody sequence to establish correct pairing of the heavy and light chains. This includes a method that relies on the in vitro Fab-arm exchange of antibodies. It relies on the separate expression of the two antibodies which will form the bispecific antibody. The two different antibodies are purified separately and subsequently combined using in vitro Fab-arm exchange to form the desired bispecific IgG-like molecule [8,9,10]. Preference for hetero- over homo-dimerization of the heavy chain is achieved by mutations in the constant region [11,12,13,14,15,16]. In a second method for bispecific antibody generation, correct pairing of the two different light chains is achieved by mutations in the heavy-light chain interface, in addition to mutations in the heavy chain constant region to yield heterodimerzation [17,18,19,20,21]. This approach allows for the expression of all four protein chains of the bispecific molecule in one cell. Finally, IgG-like bispecifics can be generated by combining two Fab arms with different specificities that carry the same light chain. Similar to the aforementioned workflows, hetero-dimerization of the two heavy chains is achieved by mutations in the constant region yielding a common light chain bispecific Ab (cLC-bsAb). The advantage of this format is that it requires the expression of only three different antibody chains—two heavy chains with different binding specificities and the common light chain—reducing complexity in therapeutic development and manufacturing. cLC-bsAbs have been approved (e.g., Emicizumab [21,22]) or are currently being evaluated in the clinic (e.g., Zenocutuzumab [23], Odronextamab [24], Linvoseltamab [25]).

Several methods have been established to discover common light chain antibodies. One approach is to generate antibody phage libraries that carry a limited light chain diversity [26,27,28]. However, for some antigens, it can be very difficult to reach the targeted antibody affinity using a displayed library approach without an additional affinity maturation step. An alternative approach to the antibody display library is animal immunization. Common light chain antibodies can be isolated from animals containing a diverse light chain immune repertoire by identifying antibody pairs that maintain binding when using the same light chain [24]. Large-scale screening can be achieved by generating antibody display libraries from immunized animals where the heavy chain repertoire is paired with a common light chain. Subsequent library selection allows for the identification of heavy chain sequences that maintain binding when paired with the common light chain [29].

Alternative sources for common light chain antibodies are genetically modified animals (Supplementary Figure S1). Several transgenic common light chain animals that produce a human antibody repertoire with a single human light chain have been described in the literature [30,31,32,33,34]. However, although all antibodies in the immune repertoire of these animals carry the same light chain in principle, the light chain undergoes somatic hypermutations (SHM) during affinity maturation. Therefore, to isolate binders from these animals, the heavy chain repertoire has to be paired with the non-mutated version of the common light chain and resulting cLC antibodies have to be screened for antigen binding. Alternatively, the heavy chain immune repertoire can be sequenced, and the identified clonotypes can be expressed and tested for binding in the presence of the non-mutated common light chain [31]. The advantage of such animals is their ability to generate fully human antibodies, which reduces the need for further engineering (e.g., humanization) and the risk of anti-drug antibodies in humans.

Here we explore endogenous common light chain mice as an alternative to humanized common light chain animals. We believe humanization of mouse antibodies nowadays poses insignificant hurdles to the antibody discovery process and minimally altered animal models such as the approach described in this work could produce a robust immune response. Several previous studies on the regulation of B cell receptor (BCR) germline rearrangement describe mouse models which contain a pre-arranged kappa light chain as a tool to investigate the B cell ontogeny including the role of surrogate light chain expression [35], receptor editing [35,36], and allelic exclusion in bone marrow and germinal centers [37]. These studies demonstrate an immune repertoire which consists of antibodies carrying a common kappa light chain. Using those design principles, we generated a minimally modified common light chain mouse strain (cLCM) which carries a pre-arranged, modified, murine IGKV10-96/IGKJ1 segment replacing the whole IGKJ locus. We first described the rationale behind the light chain locus selection for genetic engineering, and then extensively characterized the immune repertoire of the cLC mouse and demonstrated that these animals provide an alternative approach for the discovery of common light chain antibodies.

## 2. Materials and Methods

### 2.1. Mouse Model Generation

The cLCM mouse model was generated at genOway S.A. (Lyon, France). In the first step, an expression vector was constructed covering the leader exon L1, leader intron, leader exon L2, the prearranged IGKV10-96/J1-96L segment, IGKJ intron and the IGKC constant region (Supplementary Figure S2). Two versions of this construct were generated, one using the naïve sequence and one with an optimized sequence to remove potential aberrant splicing sites. Vectors were expressed in murine 3T3 cells and the resulting transcript sizes were determined by PCR amplification from cDNA. Both expression vectors yield transcripts with the expected sizes, indicating that the correctly spliced product was formed. However, an additional smaller product was observed using the construct with the naïve sequence, which was determined by sequencing to be an aberrant splice variant stemming from the 3′ of the L1 and a cryptic splice site within the prearranged segment. The absence of aberrant splicing variants in the optimized sequence construct was further confirmed by Sanger sequencing. Using the optimized expression sequence, a targeting vector was constructed which contains, in addition to L1/leader intron/L2-IGVK10-96/J1-96L segment, an additional 5′ segment and 3′ segments. The 5′ segment contains a homology region, covering a 2.8 kb region upstream of the J1 segment in wildtype C57BL/6 mice and a neomycin cassette flanked by loxP sites. The 3′ segment contains a 3.5 kb homology region covering the IGKJ intron and IGKC exon and Diphtheria Toxin cassette which serves as a negative selection marker (Supplementary Figure S3). The targeting vector was linearized and electroporated into C57BL/6 embryonic stem cells (ES), which were subject to positive and negative selection. ES clones were screened using a PCR assay which detects the 5′ genomic/transgenic junction and correct insertion was subsequently confirmed using sequencing. Random non-homologous integration was excluded by assessing the presence of the neomycin cassette in genomic DNA, which confirmed that only one integrated copy is present in the selected clones. Knock-in ES clones were injected into blastocysts resulting in chimeric animals. Breeding was established with C57BL/6 Cre deleter mice to excise the Neomycin cassette and generate heterozygous knock-in mice. The resulting offspring was screened by PCR to confirm excision of the neomycin cassette. Additional validation of knock-in and neomycin cassette excision was confirmed by sequencing the entire targeted locus in addition to 1 kb up- and downstream. Validated animals were mated to produce homozygous common light chain mice.

### 2.2. Serum Concentration of IgG, IgM and Kappa Antibodies by ELISA

Naïve serum IgM titers and IgG titers in cLCM and wildtype C57BL/6 mice were determined using an IgM mouse ELISA kit (Abcam, Cambridge, UK) and an IgG mouse ELISA kit (Abcam) following the manufacturer’s protocol. Naïve serum kappa antibody titers were determined using a Mouse Kappa Light Chain (Sandwich ELISA) kit (LSBio, Lynnwood, WA, USA) following the protocol provided by the manufacturer’s protocol.

### 2.3. Animal Immunization

For animal immunization, we utilized the model antigen ovalbumin (InvivoGen, San Diego, CA, USA) and two soluble human antigens (antigen A and B) generated specifically for inhouse projects at 23andMe. To compare the immune response of cLCM with wildtype C57BL/6 mice, three female 7-week-old animals in each cohort were immunized by weekly intraperitoneal and subcutaneous injections with 50 μg antigen (InvivoGen) and a mixture of Toll-like receptor adjuvants [38]. After four immunizations, serum from immunized mice was collected to test the antigen-specific antibody titer using ELISA. Four days before euthanization, animals received a final boost of 50 μg of antigen intravenously with no adjuvant. Mice were euthanized by carbon dioxide asphyxiation followed by cervical dislocation, as recommended by the Office of Laboratory Animal Welfare (OLAW, Bethesda, MD, USA), National Institutes of Health.

### 2.4. B Cell Phenotypic Analysis by Flow Cytometry

Briefly, single-cell suspensions from bone marrow, spleen, and peritoneal cavity lavage were isolated from immunized cLCM and, for comparison, C57BL/6 littermates. A total of 10^6^ cells were suspended in FACS buffer (1× PBS (pH 7.2), 2% FBS, 2 mM EDTA) and B cells were stained with premixed combinations of fluorochrome-labeled mAbs at concentrations optimized by titration, and total B cells were gated as singlet, live, CD19+, and/or B220+ lymphocytes. All Abs were obtained from Biolegend unless otherwise stated. The primary labeled mAbs used were AF700 or AF594 conjugated α-B220, BV510 or APC-Cy7 α-CD19 (BD Biosciences, Franklin Lakes, NJ, USA), BV605-conjugated α-IgD, Percp cy5.5-conjugated α-IgM (BD Biosciences), PE-Cy7 conjugated α-CD21, APC-Cy7-labeled α-CD23, PE Cy7-conjugated α-CD93, PE Cy7-conjugated α-CD43, AF700-labeled CD5, FITC-conjugated α-kappa, and APC-conjugated α-lambda. 4′,6-diamidino-2-phenylindole (DAPI) was used to exclude dead cells. FACS was performed using a CytoFLEX flow cytometer (Beckman Coulter, Brea, CA, USA) and data was analyzed using FlowJo v10 (Tree Star, Ashland, OR, USA) software.

### 2.5. Antigen-Specific B Cell Sorting

Spleen and lymph nodes were harvested from immunized mice five days after the final boost. B cells were enriched using theEasySep mouse pan-B cell isolation kit (Stemcell Technologies, Vancouver, CA). Cells were then stained with a fluorophore-labeled antibody panel, which includes PerCP-Cy5.5 conjugated anti-mouse IgM (BD Biosciences), PerCP-Cy5.5 conjugated anti-mouse IgD (BD Biosciences), APC-Cy7 conjugated anti-mouse CD19 (BD Biosciences), and a cocktail of phycoerythrin (PE) conjugated antibodies used during sorting as a dump channel: anti-mouse Ly6g (Biolegend, San Diego, CA, USA), PE anti-mouse CD3 (Biolegend), and PE anti-mouse F4/80 (Biolegend) and Alexa647 and Alexa488 ovalbumin (InvivoGen, San Diego, CA, USA). Single dump channel-/CD19+/IgM-/ovalbumin^duel+^-stained B cells were FACS sorted and barcoded using the Chromium controller (10× Genomics, Pleasanton, CA, USA) and a sequencing library was prepared according to the manufacturer’s instructions using the Chromium Next GEM single-cell 5′GEM, BCR amplification and library construction kit (10× Genomics, Pleasanton, CA, USA).

### 2.6. Single-Cell Sequencing and Analysis

The sequencing libraries were sequenced using an Illumina NextSeq Sequencer in conjunction with the Mid Output Sequencing Kit (Illumina, San Diego, CA, USA) ensuring at least 5000 reads per sorted cell. VH and VL sequences were assembled from the sequencing data using the Cell Ranger software version 7 (10× Genomics, Pleasanton, CA, USA). The Cell Ranger output was further analyzed to enable clone selection using R. We first filtered the data for cells containing exactly one VH and one VL sequence and clustered the paired sequences into clonotypes based on the VH germline, CDR-H3 length and the CDR-H3 sequence identity (>80% amino acid identity) using CD-HIT [39]. We further calculated for clonotypes, which consists of at least 5 clones, a consensus sequence and for each clone in those clonotypes, the hamming distance to the consensus sequence. We flagged sequences that contained sequence liabilities such as N-glycosylation sites and unpaired cysteines in the CDRs. Forty-eight clones each from the wildtype C57BL/6 and the cLCM were selected for gene synthesis and small-scale expression based on the following criteria: larger clonotype size, minimal hamming distance to clonotype consensus and no sequence liabilities in the CDR sequences. Down sampling was carried out by randomly selecting sequences from the filtered Cell Ranger output and assigning clonotypes to the down sampled dataset. Gini-coefficient and D50 diversity measures were also calculated using R.

### 2.7. Heavy Chain Immune Repertoire Sequencing and Analysis

RNA was extracted from 300,000 splenocytes derived from the ovalbumin-immunized cLCM using the RNeasy extraction kit (Qiagen, Hilden, Germany). A 5′ rapid amplification of cDNA ends (RACE) was carried out using the SMARTer^®^ RACE 5′/3′ Kit (Takara Bio, Kusatsu City, Shiga Prefecture, Japan). The resulting first-strand cDNA synthesis reaction was used as input DNA for the PCR amplification reaction. The forward primer and reverse primer mix used in the first PCR reaction is listed below. A nested PCR was carried out as described previously [40] using a reverse murine IgG primer mix which contains equal concentrations (10 µM) of the following primers: mIGG12_R2_Nested (ATTGGGCAGCCCTGATTAGTGGATAGACCGATG), mIGG12_R2.1_Nested (ATTGGGCAGCCCTGATTAGTGGATAGACTGATG), mIGG3_R2_Nested (ATTGGGCAGCCCTGATTAAGGGATAGACAGATG) and mIGGKC_R2_Nested (ATTGGGCAGCCCTGATTGGATGGTGGGAAGATG). The PCR product was cleaned up using SPRI beads (Beckman Coulter Life Sciences) and a sequencing library was prepared using NEBNext Multiplex Oligos and the NEBNext Ultra DNA Library Prep Kit (both New England Biolabs, Ipswich, MA, USA). The library was sequenced on an Illumina MiSeq Sequencer using the MiSeq Reagent Kit v3 (600 cycle) (Illumina). Paired consensus R1 and R2 reads were assembled into a single sequence spanning the VH domain using FLASH pairwise aligner [41]. IGHV, IGHD and IGHJ germlines were assigned using IgBlast [42] and the IMGT published mouse reference germline set. Data was plotted using R/ggplot2 [43].

### 2.8. Small-Scale Antibody Expression

Selected clones from the single B cell sequencing dataset were produced by DNA synthesis and transcriptionally active PCR (TAP) as chimeric human IgG1/kappa antibodies. In brief, the antibody variable-region DNA which contains 5′- and 3′-TAP-specific adapter sequences were synthesized (IDT DNA, Coralville, IA, USA). The variable region DNA, a promoter DNA fragment, and a heavy or light chain constant region DNA fragment were assembled and amplified via two rounds of overlap PCR to produce two separate linear DNA fragment products, encoding the heavy chain and the light chain, respectively. The linear heavy and light chain fragments in a 1:2 ratio were transiently co-transfected into Expi293 cells at a 3 mL scale. Cells were incubated for six days before the supernatant was harvested by centrifugation and the expressed antibodies were then purified using protein A chromatography.

### 2.9. Enzyme-Linked Immunosorbent Assay (ELISA) to Determine Antigen Binding

Microtiter plates (96-well) were coated overnight with ovalbumin (InvivoGen, San Diego, CA, USA) at 1 μg/mL, then blocked with 1% bovine serum albumin in phosphate-buffered saline with 0.05% Tween 20 (PBS-T). Antibodies (10 μg/mL) were diluted in a blocking buffer and 100 μL was added to each well. Plates were then incubated at room temperature for one hour. Then, a horseradish peroxidase (HRP)-labeled goat anti-human IgG Fc antibody (Jackson ImmunoResearch, West Grove, PA, USA) was used as the secondary reagent. The ELISA plates were developed using a TMB solution, and the reaction was stopped by the addition of 2 M H_2_SO_4_. Absorbance was read at 450 nm on a VersaMax microplate reader (Molecular Devices, San Jose, CA, USA). Antibodies that showed an absorbance higher than 0.3 OD were considered to be ovalbumin binders.

### 2.10. Biacore

Antibody association and dissociation rates were determined by Surface Plasmon Resonance (SPR) measurement using a Biacore 8K instrument (Cytiva, Marlborough, MA, USA). Each antibody at 0.5 μg/mL in the HBS-P (0.01 M HEPES, 0.15 M NaCl and 0.05% Surfactant P20) running buffer was captured by a Protein A chip (Cytiva, Marlborough, MA, USA). To measure the binding kinetics, ovalbumin (InvivoGen, San Diego, CA, USA) from 11 nM to 100 nM in 3-fold serial dilutions, and a blank buffer for baseline subtraction, were injected at 30 μL/min for 120 s, followed by a 10 min dissociation period. Regeneration of the Protein A surface was achieved via 30 s of 10 mM glycine (pH 1.5) at 50 μL/min between each running cycle. All kinetic experiments were performed at 25 °C.

### 2.11. Epitope Binning of Wildtype and cLC Antibodies

Epitope binning was performed based on a previously established protocol [44]. Biotinylated OVA was captured at a concentration of 2 ug/mL to MagAvidin beads (Luminex, ThermoFisher, Waltham, MA, USA). After washing, protein-coated beads were incubated with selected benchmark antibodies from the wildtype C57BL/6 or cLCM, at 5 ug/mL of antibody with 1.25 × 10^6^ beads/mL (one benchmark mAb per MagAvidin bead type). After a second wash, all bead types were pooled. Prior to pooling, an aliquot of each bead type was removed to serve as single bead controls. All test panel antibodies from the wildtype C57BL/6 or cLCM mice were then prepared in a 96-well half-skirted PCR plate, with each well containing antibodies at a 3.3 µg/mL concentration. Pooled beads were added to each well of the plate and incubated with the test panel antibodies. Beads were subsequently washed and the detection antibody (Jackson immunoresearch, Goat anti human-FITC) was then added. Beads were again washed and after resuspension, plates were read on a Luminex instrument. For analysis, geometric means of the fluorescence of each sample were calculated in FlowJo. Binding of each antibody relative to the reference antibody was determined by the following calculation: net flow cytometry geomean = (panel antibody and reference Ab total geomean)—reference Ab geomean. The net flow cytometry geomean values for each specific antibody across the reference panel reveals its competitive binding profile. The similarity between two antibody competitive binding profiles was calculated using the correlation coefficient (r) in Microsoft Excel and was utilized to group antibodies into epitope bins.

### 2.12. Statistical Analysis

Statistical significance was calculated using the statistical program R and the Wilcoxon rank test using adjusted *p*-values. If not otherwise noted, error bars represent standard deviation. Significance levels were labeled as follows: n.s non-significant, * ≤0.05, ** ≤0.01, *** ≤0.001, **** ≤0.0001.

## 3. Results

### 3.1. Model Generation

Previous germline rearrangement studies suggest that mouse models expressing a common light chain immune repertoire can be generated with minimal genetic engineering by following some guiding principles. First, complete replacement of the IGJK locus with the pre-arranged Vκ/Jκ segment ensures that no additional J-segments are available downstream of the inserted Vκ/Jκ segment for secondary rearrangement, which can lead to deletion of the pre-arranged segment at the kappa locus [36]. Furthermore, it would be beneficial to use a pre-arranged Vκ/Jκ segment that does not likely lead to the development of self-recognizing BCR, which would lead to receptor editing and potential replacement with a lambda light chain [35,45,46].

To select a particular prearranged IGKV/IGKJ germline combination for model generation, we identified IGKV10-96 as a frequent germline in the mouse immune repertoires [47,48,49], which promiscuously pairs with various heavy chains [48] ensuring maintenance of a diverse heavy chain repertoire. Furthermore, the amino acid sequence of IGKV10-96 does not contain any obvious sequence liabilities such as unpaired cysteine and N-glycosylation sites, minimizing potential downstream therapeutic antibody development risks. IGKV10-96 shares 75% sequence identity with human IGKV1-33 and IGKV1-27 germlines accounting for 10.5% of therapeutic antibodies (87 antibodies) that are either approved or under therapeutic development based on the TheraSabDab database [50] (Supplementary Figure S4). The high sequence simililarity with those frequently used human germlines should facilitate humanization for therapeutic antibody development. Finally, an IGKJ1 segment was chosen as it is the most frequently used J-segment in mice and most frequently paired with IGKV10-96 [49]. However, the CDR-L3 of a pre-arranged IGKV10-96/IGKJ1 segment carries a tryptophan at position 96 (Chothia numbering) which can be a sequence liability as tryptophan is prone to oxidation which may negatively impact antibody binding [51]. To reduce the chance of potential development issues for antibodies stemming from the cLCM, we replaced the tryptophan at position 96 in the pre-arranged IGKV10-96/IGKJ1 segment with leucine, a prevalent residue at position 96 in both human and mouse antibodies. We will refer to the resulting gene segment as IGKV10-96/IGKJ1-96L.

**Figure 1:**
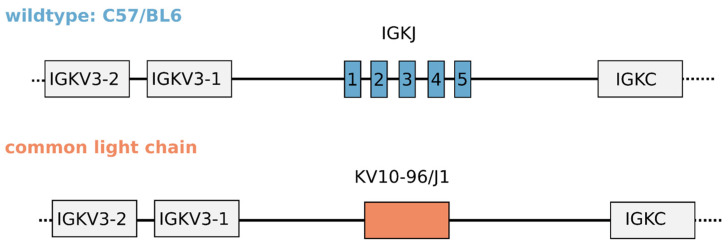
Design of the common light chain mouse model. Upper panel schematic of the kappa locus in wildtype C57BL/6 mice showing the IGKJ segments, upstream IGKV segments and downstream IGKC region. Lower panel schematic shows the same region after knocking in the prearranged IGKV10/IGKJ1 segment, which leaves the upstream and downstream regions unaltered. The schematic is not drawn to scale. See main text for detailed rationale of the model design.

The cLC mouse model was constructed in a C57BL/6 background using a knock-in approach utilizing homologous recombination in which the IGKJ cluster is replaced by the pre-arranged VK10-96/J1-96L segment (Figure 1, Supplementary Figure S3). This approach ensures that the VK10-96/J1-96L segment is embedded in its endogenous locus which should allow for transcriptional control and post-transcriptional processing similar to that for endogenous rearranged light chains.

### 3.2. B Cell Development in the Common Light Chain Mice

**Figure 2:**
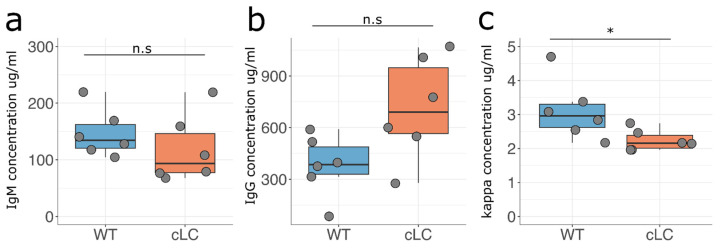
Antibody serum titers in wildtype and cLCM C57BL/6 mice. (**a**) IgM, (**b**) IgG and (**c**) kappa serum concentration of C57BL/6 mice (wildtype, blue, *n* = 6) and common light chain mice (cLC, red, *n* = 6). Measurements from individual mice are represented by a gray circle. The differences in concentration of IgM and IgG antibodies in serum of C57BL/6 mice and common light chain mice was not significant using Wilcoxon rank test. The kappa antibody concentration was determined to be significantly different (Wilcoxon rank test, * *p*-value < 0.05; n.s. not significant).

To understand if insertion of the pre-arranged IGKV10-96/IGKJ1-96L segment impacts B cell development, we measured the serum concentration of the kappa light chain, IgM, and IgG antibodies in the homozygous cLCM and compared it to wildtype (wt) C57BL/6 mice. Overall, the cLCM did not significantly differ from wildtype C57BL/6 mice in IgM and IgG antibody concentrations, while we observed a slightly lower kappa antibody serum concentration in the cLCM (Figure 2).

We then measured the immune response in the mice challenged with ovalbumin (OVA). Six weeks after immunization, similar serum antibody titers against OVA were detected in both wildtype C57BL/6 and the cLCM (Supplementary Figure S5). We also characterized the phenotype of spleen B cells in the mice challenged with OVA. Similar percentages of CD19+ B cells and OVA-specific B cells were observed in splenocytes from both wildtype and cLCM mice. In both cases, the majority of B cells (~90%) expressed the kappa LC, while reduced lambda LC expression was observed in the genetically modified mice. In addition, cells with a mature follicular phenotype (CD23+/CD21int) were also present at similar levels (>60%) in cLCM and wildtype C57BL/6 mice (Supplementary Figure S6). The data suggests a normal immune response in the cLC mouse model.

**Figure 3:**
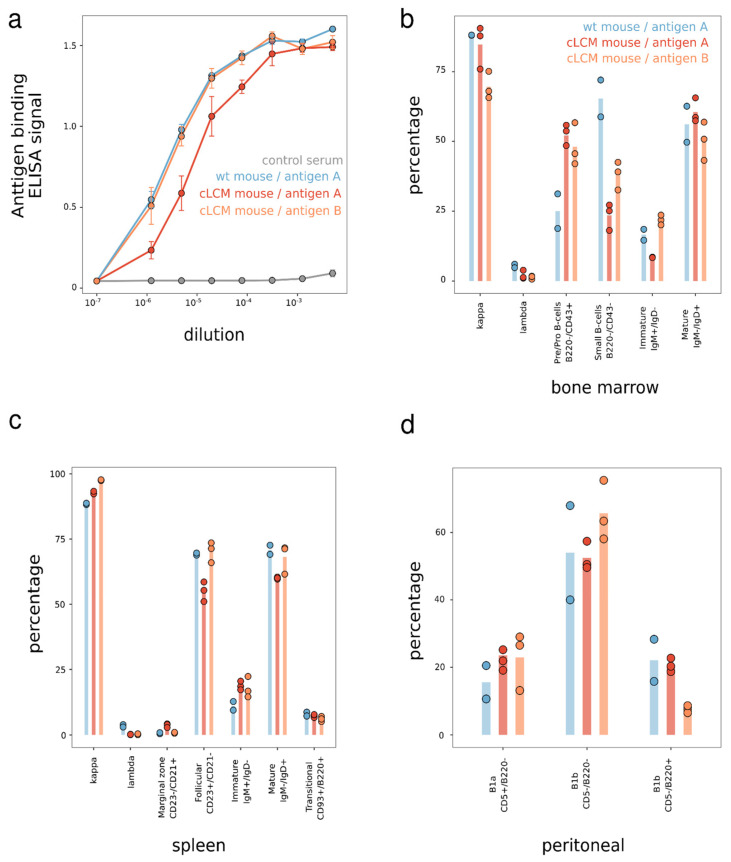
Characterization of B cell-specific immune response upon immunization; Immune repertoire characterization of immunized mice (N = 2 for the antigen A immunized wildtype mice, N = 3 for the antigen A or antigen B immunized cLC mice, respectively). (**a**) Antigen A and antigen B serum antibody titration by ELISA. The error bar represents standard deviation. (**b**–**d**) Analysis of B cell populations in each experimental group. The tissues from each immunized mice group were collected, stained, and subjected to FACS. (**b**) Percentage of B cell subpopulations in the spleen, as revealed either by surface Kappa and Lambda expression or IgM and IgD expression of single live total B cells (CD19+B220+), or transitional B cells (CD93+B220+) of single live B cells (CD19+), or mature follicular B cells (CD23hiCD21int) and marginal zone B cells (CD23intCD21hi) in total B cells. (**c**) Percentage of B cell subsets in b one marrow. FACS analysis of cells stained for CD43 and B220 expression to identify pro/pre-B cells (B220loCD43+), small pre-B (B220loCD43−), immature/mature (imm/mat B220hiCD43−) B cells. Indicated B cell subsets were pre-gated as single live CD19+ B cells analysis of surface IgM and IgD or surface L chain κ and λ expression for recirculating B cells. Indicated B cell subsets were pre-gated as single live total B cells. (**d**) Analysis of peritoneal B cell subsets as revealed by surface staining of B220 and CD5 in the pre-gated live CD19+ B cells. B cell subsets are defined as follows: B1a (B220−CD5+), B1b (B220−CD5−), B2 (B220+CD5−), and the percentage of each subset was indicated in the graph.

To further examine the cLCM’s immune response to antigens other than OVA, we characterized the B cell development in the cLCM challenged by two antigens in another cohort study (Figure 3). In this study, two wildtype C57BL/6 mice were immunized with one human protein antigen (antigen A) and six cLCMs were immunized with two different human protein antigens (antigen A and antigen B). Robust immune response was observed in the cLC mice challenged with both antigens (Figure 3a). B cell development was evaluated by comparing the splenic, bone marrow, and peritoneal B cell compartments in both C57BL/6 and the cLCM. We observed that ~90% of spleen B cells express a kappa light chain and that 50–70% of B cells are mature follicular (CD23+/CD21int) B cells among all immunized mice splenocytes (Figure 3b). The presence of mostly mature B cells is also observed using (IgM^low^/IgD^high^) markers. In addition, we observe a similar percentage (6–8%) of transitional B cells (CD93+/B220+) in the spleen across all cohorts.

To further investigate if the IGKV10-96/IGKJ1-96L has a negative effect on B cell ontogeny, we studied early B cell compartments in bone marrow by flow cytometry with specific surface markers as shown in Figure 3c. The B220+/CD43+ pre-B population was slightly increased, and conversely, the small pre-B cell population was decreased, while the immature and mature B cells were not affected. The total B220+/IgD+ recirculating mature B cells were similarly present in both the cLCM and wildtype mice, and most recirculating B cells expressed the kappa LC. Overall, there is no gross B cell developmental arrest evident in the bone marrow of the cLCM. Finally, B1 lymphocytes, a distinct innate-like subset of B cells, appeared to be unaffected in the genetically modified mice, as observed by CD5+/B220− staining of peritoneal cavity B cells (Figure 3d).

### 3.3. Immune Repertoire—Isotype and V Gene Usage

To compare the antibody repertoire of the cLCM with wildtype C57BL/6 mice upon ovalbumin (OVA) immunization in detail, we isolated IgM-CD3−OVA+ lymphocytes and splenocytes from pooled tissue of six cLCMs and six wildtype mice, respectively, using FACS (Supplementary Figure S7). We then obtained the paired heavy and light chain variable region sequences of the BCR from the two pools by single-cell sequencing. In total, we observed 3859 heavy and 3459 light chain sequences from the cLCM tissue from 3915 cells of which 2884 cells had exactly one heavy and one light chain pair. B cells from wildtype C57BL/6 mice yield 6149 heavy and 6427 light chain sequences from 6173 cells of which 2884 cells contained one heavy and one light chain pair.

**Figure 4:**
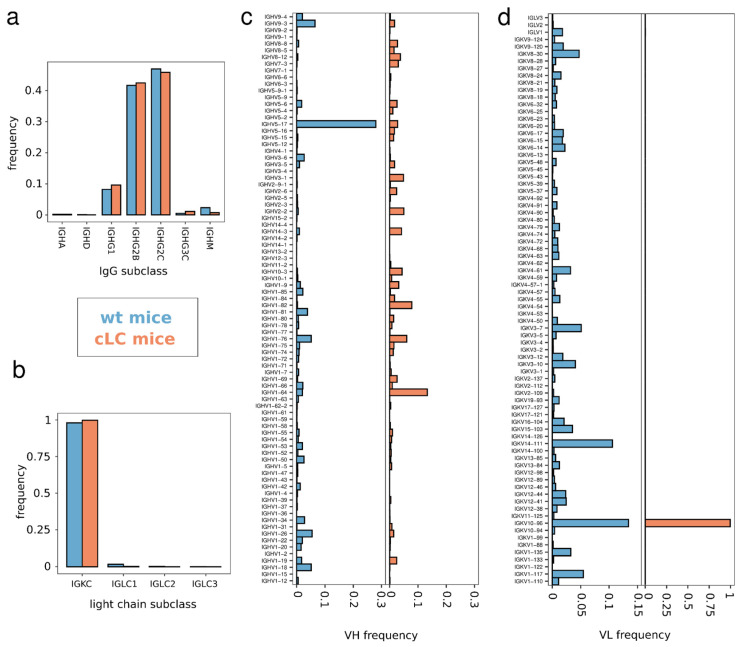
IgG subclass and germline usage of ovalbumin-specific antibodies; (**a**) subclass and IgG isotype distribution in the sequenced single-cell immune repertoire of OVA-immunized wildtype C57BL/6 mice (blue, *n* = 6089) and common light chain mice (red, *n* = 3716). (**b**) Light chain subclass distribution in the sequenced single-cell immune repertoire of OVA-immunized wildtype C57BL/6 mice (blue, *n* = 6409) and common light chain mice (red, *n* = 3394). (**c**) IGHV distribution in the sequenced single-cell immune repertoire of OVA-immunized wildtype C57BL/6 mice (blue, *n* = 6149) and common light chain mice (red, *n* = 3859). (**d**) Light chain V-gene usage distribution in the sequenced single-cell immune repertoire of OVA-immunized wildtype C57BL/6 mice (blue, *n* = 6427) and common light chain mice (red, *n* = 3429).

The Ig subclass distribution of the sequenced antibodies of the cLCM matches with the one obtained from wildtype mice (Figure 4a). Both OVA+ repertoires are dominated by IgG antibodies, the majority being of the IgG2B and IgG2C isotypes, while approximately 10% of the antibodies belong to IgG1. Apart from IgG, some IgA, IgD and IgM antibodies were observed. Light chain subclasses in both repertoires are dominated by IGK chains (Figure 4b). The percentages of lambda chain antibodies are low in both animals (2% in the wildtype pool versus 0.02% in the cLCM pool). In summary, the heavy and light chain subclass analysis supports our FACS staining results (Figure 3) demonstrating that there is no large difference in B cell development in the cLCM compared to C57BL/6 wildtype mice.

We next analyzed the VL gene usage in the two pooled repertoires to understand if the repertoire in the cLCM is indeed restricted to the prearranged IGKV10-96/J1-96L segment. The wildtype light chain immune repertoire contains antibodies which are derived from 79 different Vκ genes and 3 Vλ genes (Figure 4d). The most frequent Vκ genes are IGKV10-96 (13.4%), IGKV14-111(10.6%) and IGKV1-117 (5.4%). In contrast, the light chain repertoire of the cLCM is dominated by light chains derived from IGKV10-96 (99.7%). The result validates our cLCM model design and choice of our prearranged IGKV10-96/J1-96L segment demonstrating successful light chain expression with minimal receptor editing as evident from the absence of any other IGKV chains and a very low percentage of IGLV derived-antibodies.

Given that the cLCM repertoire consists only of IGKV10-96-derived light chains, we questioned if the use of a single light chain impacts the heavy chain diversity. Interestingly, we find differences in the VH usage between the two repertoires (Chi-squared test *p*-value: 3.163 × 10^−5^). While the C57BL/6 wildtype repertoire is dominated by antibodies derived from IGHV5-17, the most utilized VH germline in the cLCM repertoire is IGHV1-64 (Figure 4c). We observed 79 VH germlines in the wildtype repertoire and 76 VH germlines when the wildtype repertoire is randomly subsampled to the same size as the cLCM repertoire (which consists of 67 VH genes). However, using the Gini coefficient as a measure of equality of VH gene usage shows that the distribution is similar between the subsampled wildtype repertoire and the cLCM repertoire (0.72 and 0.74, respectively).

We further sequenced the VH portion of B cells from the spleen and lymph nodes of the OVA-immunized cLCM using an amplicon sequencing approach (Supplementary Figure S8). While this approach does not preserve heavy–light chain pairing and has a higher error rate due to the absence of barcodes, it allows us to obtain a deeper sequencing depth and comprehensively capture the VH gene usage of OVA+ and OVA− B cells in lymphocytes and spleen. In addition to the 67 VHs observed in the single-cell sequencing approach, we identified 27 additional VH genes in the VH-only repertoire sequencing dataset (two VH genes are unique to the 10x dataset). Overall, the data suggests that even though the cLCM repertoire is restricted to one Vκ germline, a diverse heavy chain repertoire is maintained as we observe VH usage from all VH families. In addition, a comparison between the VH gene usage of OVA+ antibodies from the cLCM and wildtype mouse suggests that the cLCM can generate an antigen-specific heavy chain repertoire that is only slightly more restricted in terms of VH usage compared to a repertoire observed in C57BL/6 wildtype mouse.

### 3.4. Immune Repertoire—Clonotype Diversity

**Figure 5:**
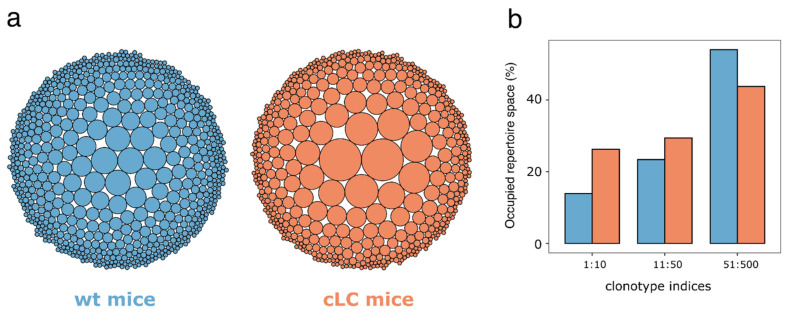
Clonotyping of ovalbumin-specific antibodies. (**a**) Bubble plot of clonotype size distribution in OVA-immunized wildtype C57BL/6 mice (blue) and common light chain mice (red). The bubble size corresponds to the relative size of the clonotype (i.e., the number of clones which make up a clonotype). (**b**) Bar graph visualizing the percentage of the sequenced immune repertoire, which is occupied by the 10 largest, the 11th to 50th largest, and the 51st to 500th largest clonotype in the OVA-immunized wildtype C57BL/6 mice (blue) and common light chain mice (red).

A diverse immune repertoire is crucial for the suitability of the cLCM for antibody discovery, as more diverse clones may result in high epitope diversity and faster drug development as clones with potential liabilities may be avoided. To investigate the diversity of the immune repertoire on a clonal level, we performed a clonotype analysis of the IgM-CD3−OVA+ sorted heavy/light chain single-cell sequence repertoires. Using only cells which contain one heavy chain and one light chain sequence, we grouped clones derived from identical VH germlines and possessing an equivalent CDR-H3 length with a high sequence identity into the same clonotype (see Section 2 for details). As the wildtype C57BL/6 mouse immune repertoire contains significantly more sequences, we down sampled the repertoire to the size of the cLCM for the subsequent statistical analysis. We identified 704 clonotypes in the wildtype repertoire which compares with 515 clonotypes in the down sampled cLCM repertoire suggesting a higher diversity of the wildtype repertoire at the clonal level. This observation is in line with a visual inspection of the clonotype distribution depicting that the down sampled cLCM repertoire contains larger clonotypes compared to the wildtype repertoire (Figure 5a). Overall, the cLCM immune repertoire is skewed to larger clonotype sizes, for example the top ten clonotypes occupy 26% of the immune repertoire in contrast to 14% in the wildtype repertoire (Figure 5b). Another common diversity metric that measures the number of clonotypes that occupy 50% of the immune repertoire (D50) confirms that the cLCM immune repertoire has lower diversity. The D50 for the cLCM is 39 in contrast to 91 for the wildtype repertoire. In summary, while the clonotype diversity of cLCM is lower than that in wildtype mice, the identification of more than 515 potential antigen-positive clonotypes in the cLCM suggests that the model is suitable for antibody discovery.

### 3.5. Light Chain Mutation Profile

**Figure 6:**
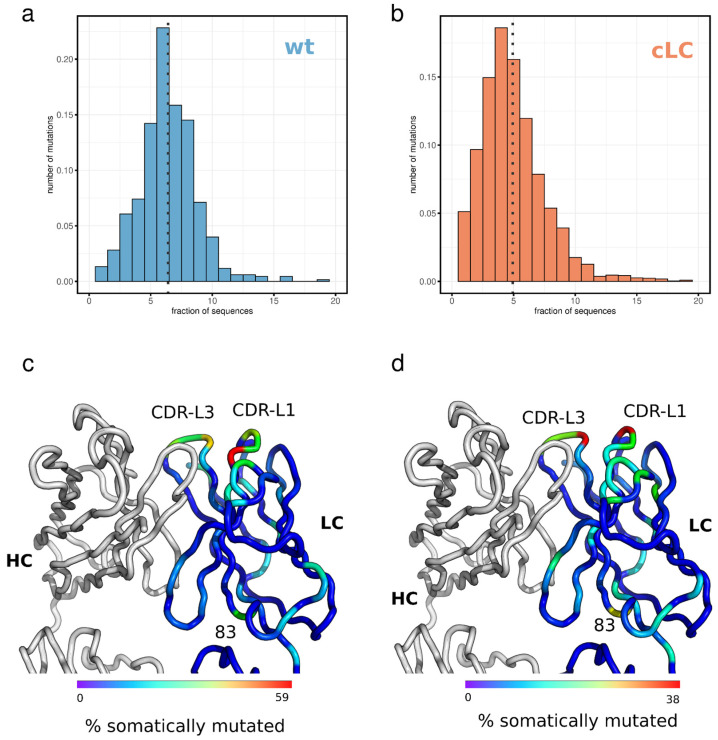
IGKV10-96 light chain variable domain mutation profiles; (**a**) distribution of amino acid mutations observed in OVA-immunized C57BL/6 mice in 675 IGKV10/IGKJ1-derived antibodies. (**b**) Distribution of amino acid mutations in the light chain of OVA-immunized common light chain mice (3420 sequences). (**c**,**d**) Structural locations of frequently mutated amino acid positions are mapped onto a structure of an IGKV10/IGKJ1 light chain (PDB code 5DO2). Positions which are mutated with higher frequency in IGKV10/IGKJ1-derived antibodies of wildtype C57BL/6 mice (**c**) or common light chain mice (**d**) have a wider cartoon size and are colored in yellow or red color, while positions which are conserved and are not frequently mutated are colored in blue.

For antibody discovery, it is essential to understand the mutation profile of the light chain. Although the cLCM immune repertoire is dominated by clones derived from a single pre-arranged Vκ/VJ combination (Figure 4d), the B cells in the cLCM will undergo affinity maturation which will likely result in the accumulation of SHMs in the common light chain. Especially, if those mutated positions are involved in antigen binding, pairing an identified heavy chain with a non-somatically mutated light chain (cLC) might result in a loss of affinity compared to the endogenous light chain. To investigate the degree the light chain accumulates SHMs, we analyzed the mutation profile of the light chain at the amino acid level using the single-cell repertoire data from the cLCM (Figure 6). We compared the mutation profile of the light chain of the cLCM to the immune profile of IGKV10-96/J1 light chains from wildtype C57BL/6 mice as light chains derived from this germline combination (IGKV10-96/J1) constitute approximately 10% of the wildtype immune repertoire. Interestingly the cLCM light chain repertoire contains on average significantly fewer somatic mutations which result in an amino acid change compared to the IGKV10-96/J1 chains in the wildtype repertoire (4.9 and 6.4 mutations per light chain, respectively; Wilcoxon rank-sum test *p*-value < 2.2 × 10^−16^) (Figure 6a,b).

The amino acid positions that are mutated by SHM are similar between the cLCM and the wildtype mice. CDR-L1 positions 30–32 and CDR-L3 positions 92–94 are mutation hotspots in the CDRs and position 83 is a mutation hotspot in the framework region (Figure 6c,d, Supplemental Figure S9). The result of the above-described mutation frequency and profile is that 67% of all CDR-L1, 61% of all CDR-L2 and 41% of all CDR-L3 in the cLCM repertoire contains at least one somatic mutation that results in an amino acid change, thus only 8% of all light chains contain no mutations in the CDR-L loops. These results suggest that for most clones, the light chain is potentially involved in antigen binding and pairing the heavy chain with the common light chain could impact affinity for the antigen.

### 3.6. Identification of OVA-Specific Antibodies

**Figure 7:**
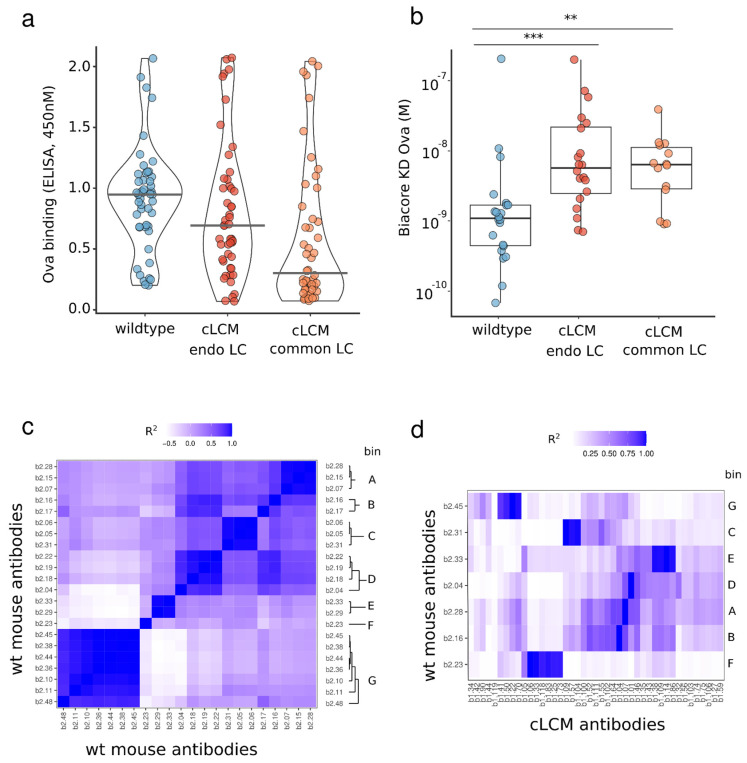
Characterization of ovalbumin binding properties of discovered antibodies. Ovalbumin binding of antibodies isolated from wildtype C57BL/6 and cLCM animals. Ovalbumin binding was assessed for 48 anti-ovalbumin clones selected from wildtype C57BL/6 mice and 48 anti-ovalbumin clones discovered from LCM with their respective endogenous light chain paired with the common light chain. (**a**). ELISA screening of ovalbumin binding (**b**). The binding affinity (K_D_) of ELISA-positive antibodies for ovalbumin is determined by Biacore 8K. Differences in binding KD were assessed using a Wilcoxon rank test. The KDs of the wildtype C57BL/6 clones were significantly different from the KDs of cLCM clones when paired with their endogenous or common light chain (adjusted *p*-value 0.00064 and 0.0032, respectively; ** indicate *p*-values < 0.005, *** indicate *p*-values < 0.001). (**c**) Epitope binning of 22 anti-ovalbumin antibodies isolated from wildtype C57BL/6 mice. Heatmap represents R^2^ values of the competitive binding profile of antibody pairs. A dark blue color represents high correlation between binding profiles of two antibodies while a light blue color represents a low correlation. The right *Y*-axis contains assigned epitope bins (A–G) based on the clustered R^2^ values. (**d**) Epitope binning of seven benchmark anti-ovalbumin antibodies isolated from wildtype C57BL/6 mice, representative of the seven identified epitope bins, against 43 cLC antibodies. A dark blue color represents high correlation (R^2^) between binding profiles of two antibodies while a light blue color represents a low correlation.

We compared the OVA-specific antibodies obtained from the cLCM and wild-type animals to provide further evidence supporting the cLCM as an effective tool for antibody discovery. Forty-eight clones from the OVA+ cLCM and from the wildtype B cell repertoire were selected for small-scale antibody expression. In both groups, antibodies were paired with their respective endogenous light chains; thus, the light chain of antibodies from the cLCM contained somatic mutations. In the initial screening step, successful antibody expression and binding to ovalbumin was confirmed by ELISA (Figure 7a). Of the 48 antibodies from wildtype mice tested for expression, 47 antibodies showed sufficient expression levels, and 42 antibodies demonstrated binding to OVA in ELISA. Of the 48 antibodies from the cLCM tested, 43 antibodies showed expression and 39 antibodies showed OVA binding in ELISA. We further characterized the kinetics and binding affinity of the OVA-specific antibodies using Biacore (Figure 7b). Twenty-two antibodies from wildtype mice and 20 antibodies from the cLCM showed binding to OVA in Biacore, exhibiting binding affinities (KD) ranging from 64 pM to 191 nM and 600 pM to 180 nM in the wildtype and cLCM group, respectively. Overall, the antibodies obtained from the cLCM had lower median affinity than the antibodies obtained from the wildtype mice (Wilcoxon rank test, *p*-value 0.00064).

Finally, we directly compare the binding activity of the cLCM antibodies we obtained using the endogenous light chain (containing somatic mutations) with the binding activity of the same antibodies using the common light chain (no somatic mutations). The 48 antibodies from the cLCM that were initially paired with their endogenous light chain, were paired with the common light chain. All of the 48 cLC clones expressed, and 23 out of 48 clones (~50%), were confirmed to bind OVA by ELISA, compared to 90% (39 out of 43) of antibodies which retained binding when paired with the endogenous light chain. The cLC antibodies which retained antigen binding in ELISA were further analyzed on Biacore. Fourteen antibodies showed binding to OVA in Biacore with binding affinities (KD) ranging from 850 pM to 120 nM. There was no significant difference in binding affinity compared to those antibodies paired with their endogenous light chain (Figure 7b, Wilcoxon rank test). The results demonstrate that although some clones lose binding after being paired with the common light chain, we were still able to obtain a set of high-affinity common light chain antibodies from the cLCM.

### 3.7. Epitope Binning of Wildtype and cLC Antibodies

Besides affinity, another important metric to evaluate the cLCM’s suitability for therapeutic antibody discovery is the epitope diversity of the antibody discovered. Larger epitope diversity increases the chances of discovering antibodies that exhibit the desired activity. To evaluate the epitope bin coverage of our anti-OVA antibodies, we carried out two separate epitope binning experiments. The initial experiment binned 24 anti-OVA antibodies obtained from wildtype mice to determine the epitope diversity obtained from these animals. Using a FACS-based binning approach, we identified eight distinct epitope bins, bins A through G (Figure 7c). We then proceeded with one wildtype anti-OVA antibody from each of the seven respective bins to serve as benchmark antibodies in our follow-up cLCM epitope binning experiment. In the follow-up experiment, we used a panel of 43 cLC anti-OVA antibodies against the seven benchmark antibodies obtained from wildtype C57BL/6 mice. We found that approximately half, (21 out of 43), of the cLC anti-OVA antibodies bin with one of the seven benchmark antibodies from the wildtype mice. The remaining 22 cLC antibodies appear to fall into new epitope bins (Figure 7d). These results demonstrate that the cLCM is able to generate antibodies against similar epitopes as their wildtype counterparts.

## 4. Discussion

The work presented here demonstrates that an endogenous common light chain mouse model, generated with minimal genetic engineering is suitable for common light chain antibody discovery. While the model presented here uses a genetic engineering approach similar to those in previously described mouse models that have been used to investigate B cell development, those previous models are not suitable for common light chain discovery as they deliberately express autoreactive light chains or light chains with a particular antigen specificity [35,45,46]. Instead, we chose the light chain gene segment IGKV10-96, because it is one of the most commonly used light chains, has been shown to pair well with a wide range of mouse VH genes without much bias and has high homology with the human kappa light chain frequently used in therapeutic antibodies. In addition, we modified the endogenous tryptophan at position 96 to leucine to reduce the likelihood of developability issues. We demonstrated that our cLCM model generates a robust immune response upon immunization as evidenced by a high antigen titer (Figure 3a and Supplementary Figure S5), normal B cell development (Figure 3b–d and Supplementary Figures S6 and S7), a diverse heavy chain repertoire with only slightly reduced VH gene usage (Figure 4) and a clonotype diversity comparable to wildtype C57BL/6 mice (Figure 5). Moreover, the vast majority of antibodies in the cLCM immune repertoire carry a light chain derived from the prearranged IGKV10-96/J1L segment while the percentage of lambda chain antibodies is low, suggesting that the expression of the selected prearranged IGKV10-96/J1-96L segment results in low receptor editing (Figure 4). Furthermore, the cLC antibodies isolated from the OVA-immunized cLCM exhibit a high affinity to the model antigen (Figure 7a,b). Finally, the OVA epitope diversity of the isolated cLC antibodies matches the diversity of anti-OVA antibodies isolated from wildtype C57BL/6 mice (Figure 7c,d). When combined with a robust antibody discovery workflow using either single-cell sequencing as presented here or alternatively workflows such as immune library display or VH repertoire sequencing, the cLCM allows for the rapid generation and identification of diverse high-affinity common light chain antibodies. If human antibodies are required, those antibodies can be humanized in bulk using established [52] or novel humanization approaches [53].

While we demonstrate that B cell development is not impaired in the cLCM, B cells in cLCMs differ from wildtype C57BL/6 B cells as they are homozygous for the rearranged IGKV10-96/J1-96L locus. As a result, both alleles of the prearranged IGKV10-96/J1 are expressed [46]. In wildtype B cells allelic exclusion prevents the simultaneous rearrangement of both kappa clusters [37,46]. However, due to differences in epigenetic modification, one allele will likely undergo SHM at higher frequency than the other resulting in the expression of two distinct BCRs on the surface of the cLCM B cells [37]. The dual allelic expression does not impact B cell development in general (Figure 3) [37], however, it has to be taken into account when interpreting the somatic mutation data (Figure 6a,b) as those data likely represent a mixture of both alleles. Therefore, the lower average mutation frequency in the cLCM is likely a product of dual allelic expression and the technical limitations in separating the two highly similar light chain alleles during single-cell sequencing.

Nevertheless, the IGKV10-96/J1 mutation profile between the cLCM and the C57BL/6 mice is similar. Most SHM occur in the CDR-L1 and CDR-L3 loops. The most common SHM in the framework is position 83 (Figure 6c,d, Supplementary Figure S9). The mutation profile suggests that the cLCM light chain is involved in antigen binding in most of the antibodies. The highly mutated positions in the CDR-L1 (pos 30–31) and in CDR-L3 (pos 92–94) are usually solvent exposed in IGKV10-96 light chains (for example, in an antibody structure with an IGKV10-96-derived light chain (PDB entry 5DO2), the relative solvent accessible surface area is between 14 and 65% for CDR-L3 position 92–94 and 25–30% for CDR-L1 position 30–31), suggesting that they directly interact with OVA. The framework position 83, while located away from the antigen, influences the overall dynamic of the Fab fragment. A change in residue at this position can influence the Fab elbow angle as well as the orientation of the VH and VL domains towards each other and has been reported to influence antibody affinity and stability [54]. This position is also optimized in human antiviral broadly neutralizing antibodies [55,56]. The direct involvement of the light chain in antigen binding and the importance of somatic mutations is demonstrated by the reduction of a binding signal when heavy chains are paired back to the non-mutated common light chain (Figure 7a,b).

Previously published approaches which detail the generation of common light chain antibodies include a humanized common light chain rat [31] and chicken [32] and common light chain phage libraries [27,28]. While a direct comparison between these different approaches can only be achieved in a side-by-side experiment as factors like antigen attributes and differences in immunization strategy influences the properties of the identified antibodies, the isolated common light chain OVA antibodies from our cLCM have a similar affinity range (KD at 0.85–120 nM) as anti-PGRN antibodies isolated from a common light chain chicken (cLCM-derived anti-OVA antibodies median KD is 5.95 nM and common light chain chicken median KD is 10.3 nM) [32], while the affinities of 4-1-BB antibodies isolated from the phage library have an initial lower mean affinity (mean KD 128 nM) but reach similar affinity after affinity maturation (mean KD of affinity matured 4-1-BB antibodies derived from the phage library is 13 nM which compares to a mean KD of the cLCM-derived OVA antibodies of 8 nM, respectively) [28]. Overall, the cLCM model presented in this work should complement the current available toolset to generate common light chain antibodies for the construction of multi-specific formats especially when phage panning is not feasible or when diversification of binders is required as the immune repertoire of humanized mice and of the cLCM will differ in CDR composition [57].

## Data Availability

Data are contained within the article and Supplementary Materials. The paired sequencing data of OVA+ B-cells from wildtype C57BL/6 and cLCM as well as the unpaired repertoire sequencing data of cLCM have been deposited in the NCBI SRA (Project number PRJNA1011966).

## Supplementary Materials

The following supporting information can be downloaded at: https://www.mdpi.com/article/10.3390/antib13010014/s1, Figure S1: Outline of a common light chain discovery workflow; Figure S2: Expression vector layout; Figure S3: Light chain V-gene usage in antibodies under clinical development; Figure S4: Mouse model engineering schematic; Figure S5: Ovalbumin-specific serum titers; Figure S6: Phenotypic comparison of B-cells in naive and ovalbumin-immunized mice; Figure S7: Sorting strategy to isolate OVA+ B-cells; Figure S8: IGHV gene usage in the antibody repertoire of cLCM; Figure S9: Observed frequency of nonsense mutations in IGKV10-96 light chains.
