# Supplementary material for: An Engineered Mouse Model That Generates a Diverse Repertoire of Endogenous, High-Affinity Common Light Chain Antibodies
Source: Antibodies (Basel). 2024 Feb 8;13(1):14. doi: 10.3390/antib13010014 (PMC10885109; doi:10.3390/antib13010014)
Supplement: Supplementary file 1 [file antibodies-13-00014-s001.zip › antibodies-2706021-supplementary.pdf]

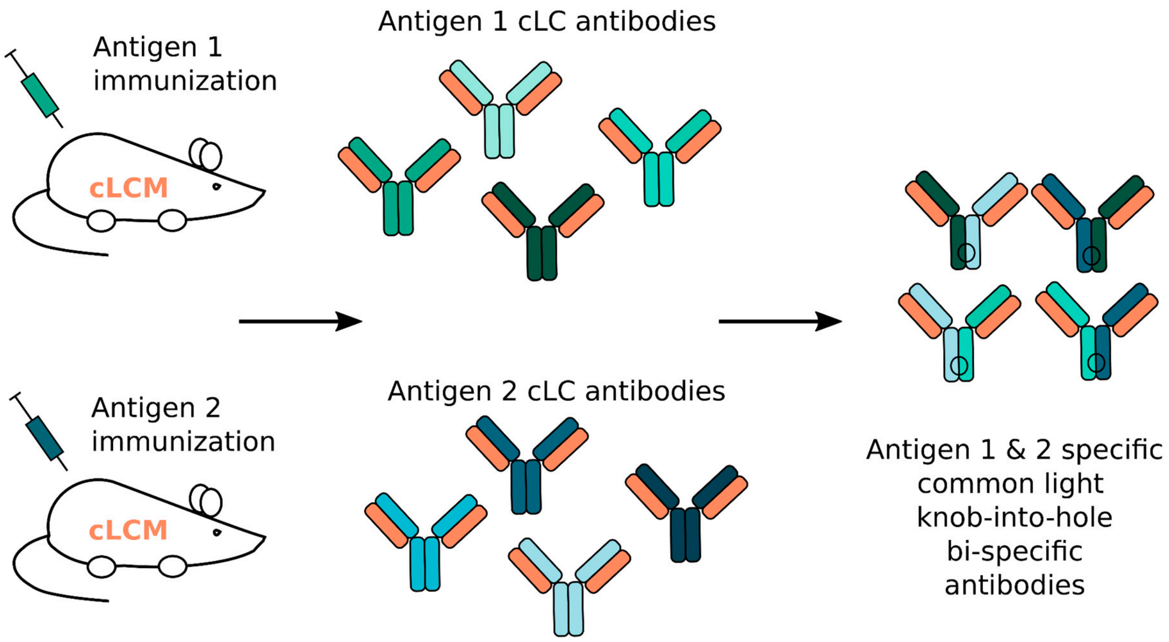

Supplementary Figure S1: Schematic outline of a common light chain discovery workflow utilizing a common light chain mouse model (cLCM). Two separate immunization campaigns against two different antigens (1 & 2) yield two common light chain immune repertoires specific for the two antigens. In a second step, bi-specific antibodies are generated by combining common light chain antibodies with different specificities into one molecule using a knob-into-whole antibody format.

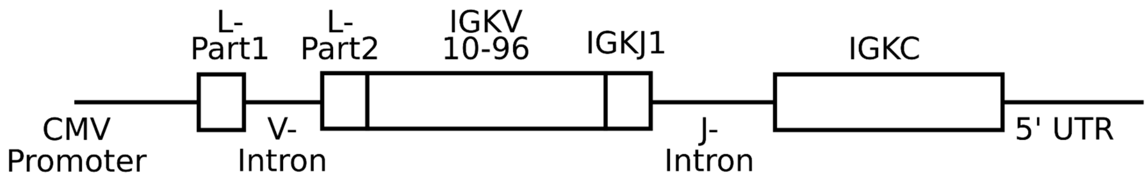

Supplementary Figure S2: Layout of the expression vector to test for correctly spliced transcript. See material and methods for details.

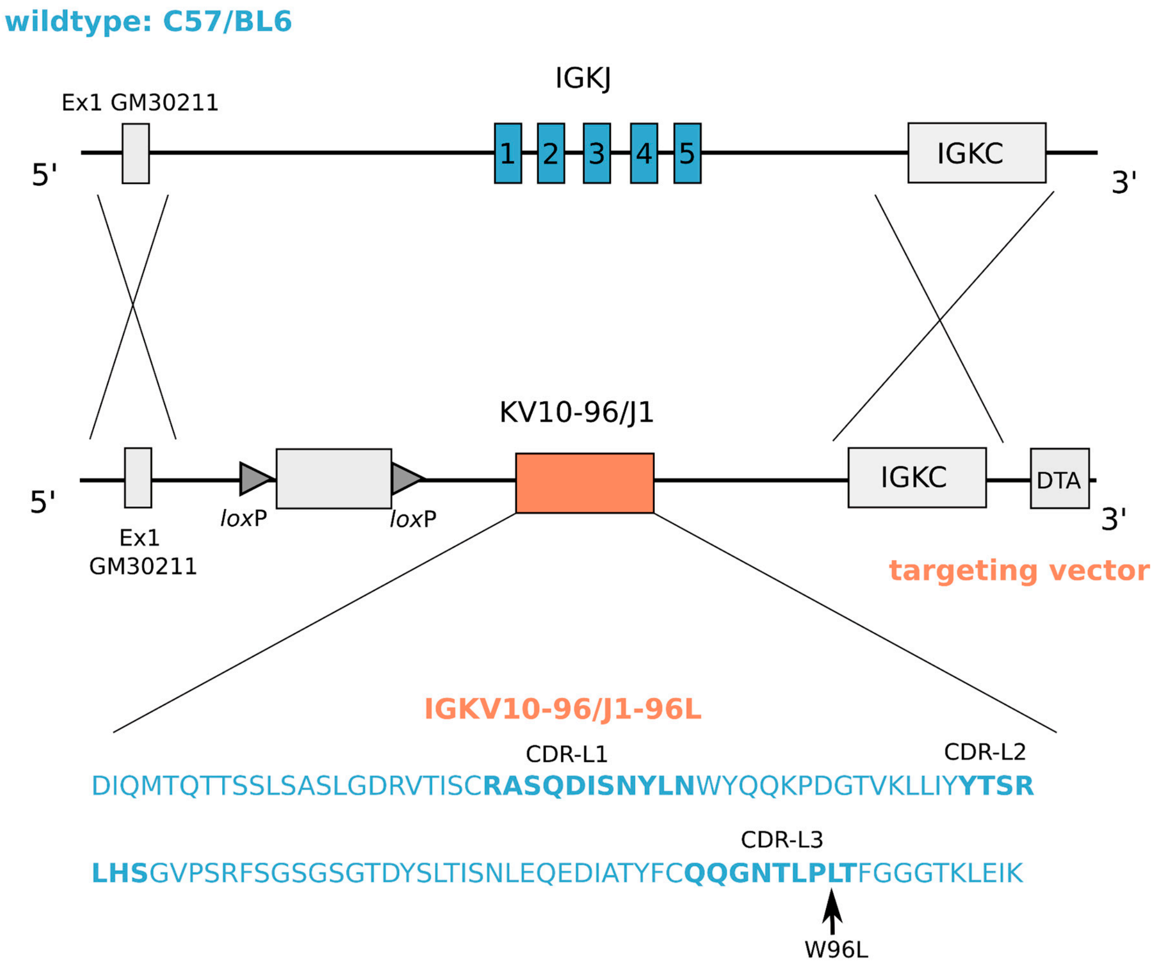

Supplementary Figure S3: Schematic showing the process to generate the common light chain mouse model by knock-in via homologous recombination.

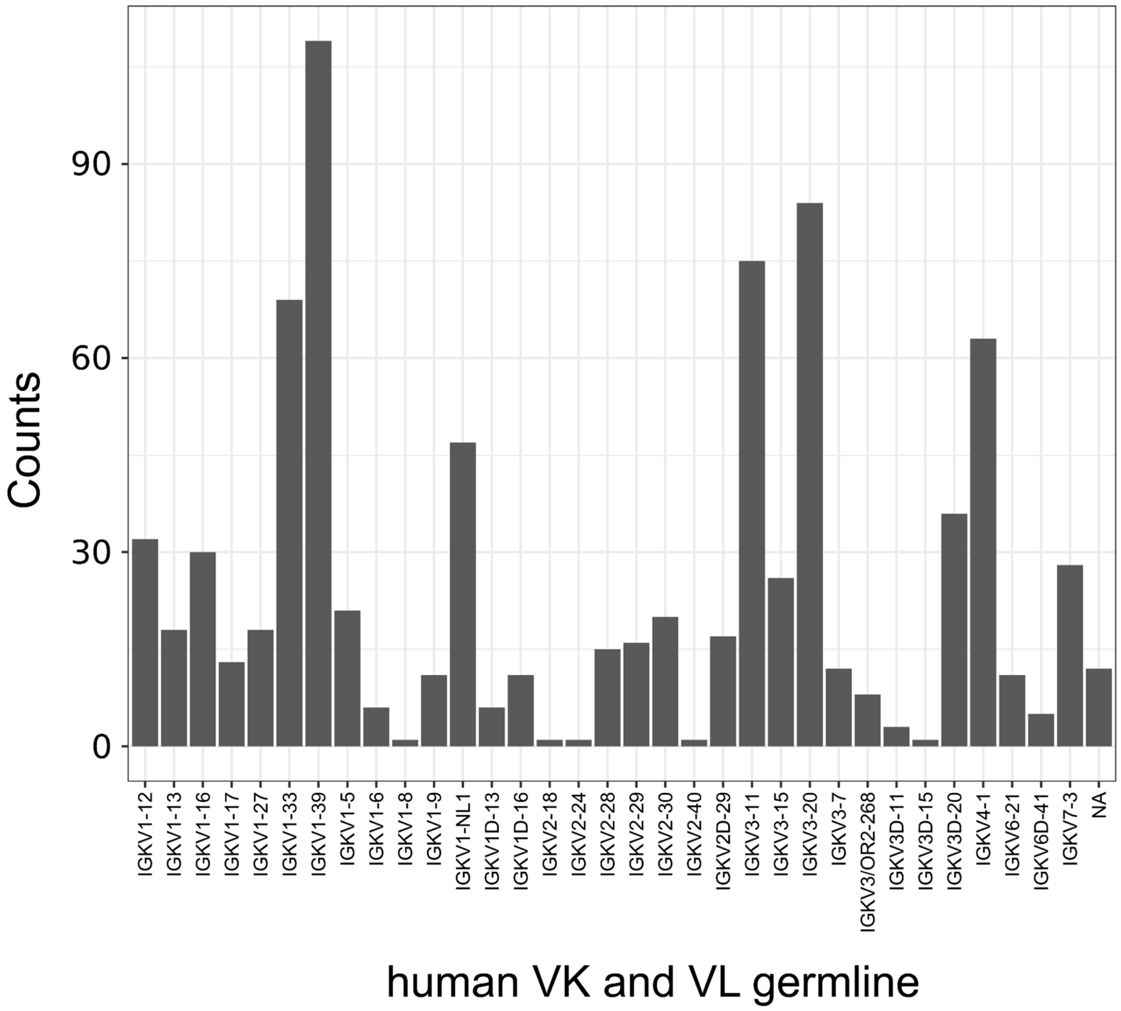

Supplementary Figure S4: Bar graph showing the light chain V-gene usage in antibodies which have been approved or are currently under clinical development.

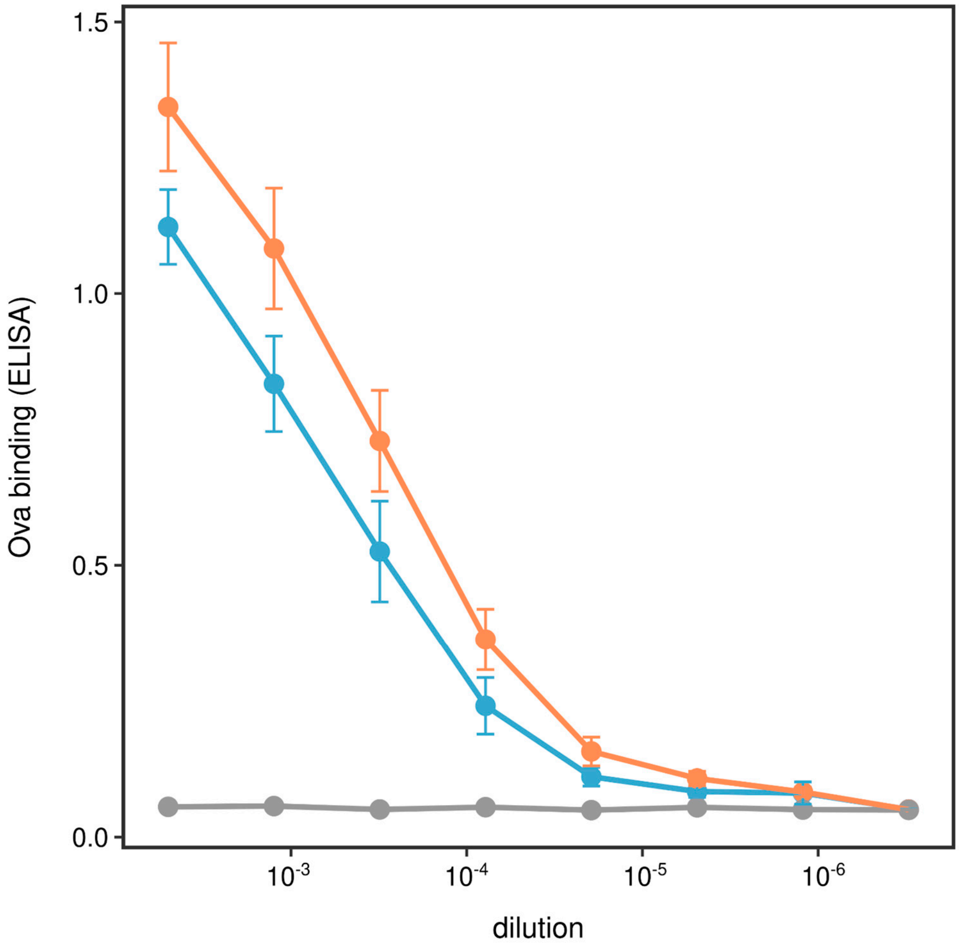

Supplementary Figure S5: Titration using ELISA to assess ovalbumin binding of serum from wildtype C57BL/6 (blue) and cLCM animals (red) immunized with ovalbumin.

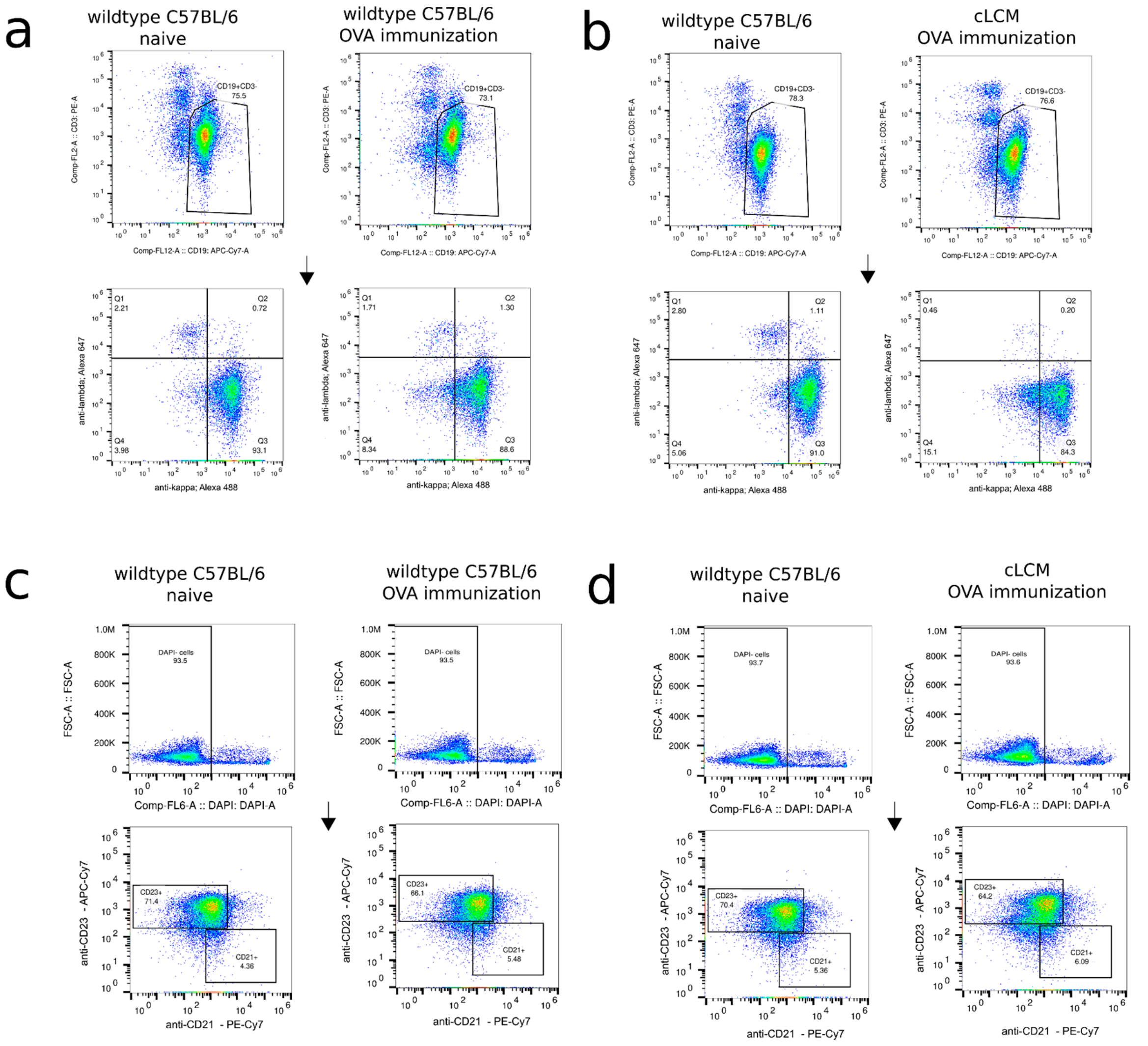

Supplementary Figure S6: Phenotypic comparison of B-cells in naive and ovalbumin immunized mice. (a.) Comparison of staining of CD19+ /CD3- B-cells for lambda and kappa chain in naive wildtype C57BL/6 and wildtype C57BL/6 immunized with ovalbumin. (b.) Comparison of staining of CD19+ /CD3- B-cells for lambda and kappa chain in naive wildtype C57BL/6 and cLCM immunized with ovalbumin. (c.) Comparison of staining for CD21 and CD23 markers in naive wildtype C57BL/6 and wildtype C57BL/6 immunized with ovalbumin. (d.) Comparison of staining for CD21 and CD23 markers in naive wildtype C57BL/6 and cLCM immunized with ovalbumin.

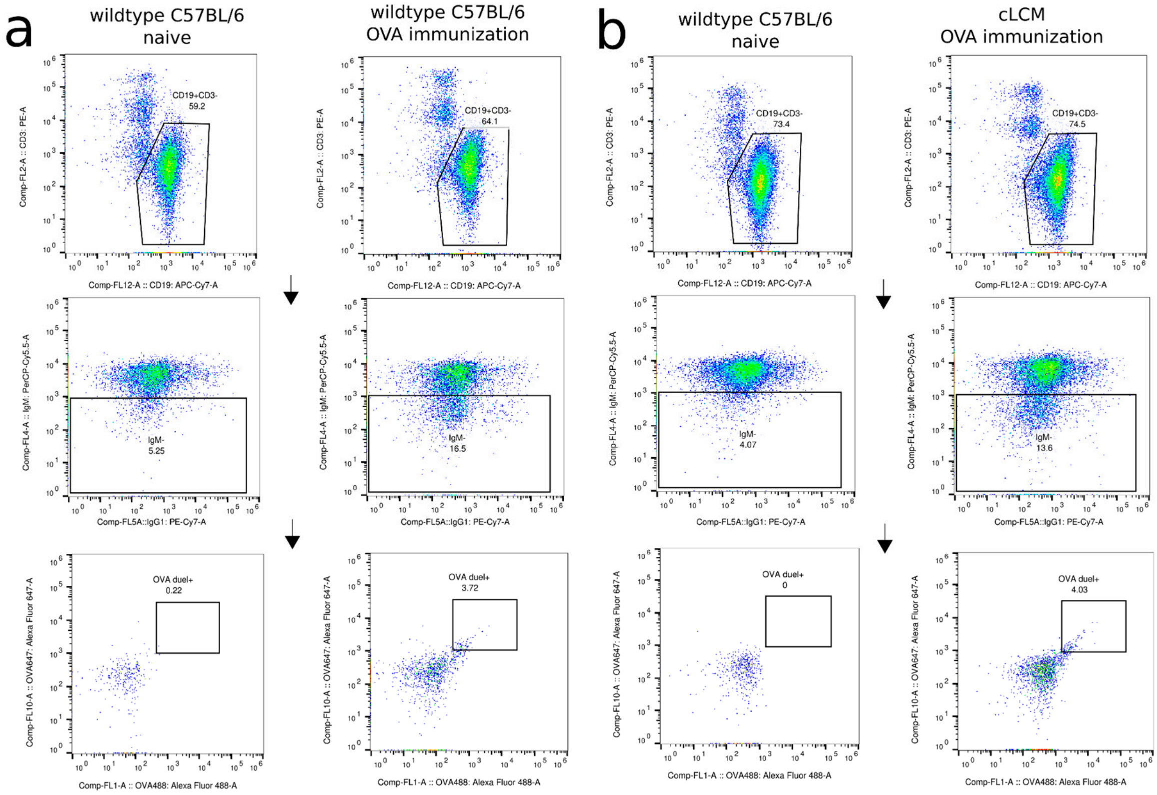

Supplementary Figure S7: Sorting strategy to isolated CD19+/CD3-/IgM-/OVA+ B cells from (a.) wildtype C57BL/6 mice and (b.) cLCM immunized with ovalbumine. Specificity of the sorting gates is validated using CD19+/CD3-/IgM- B cells from naive C57BL/6 animals.

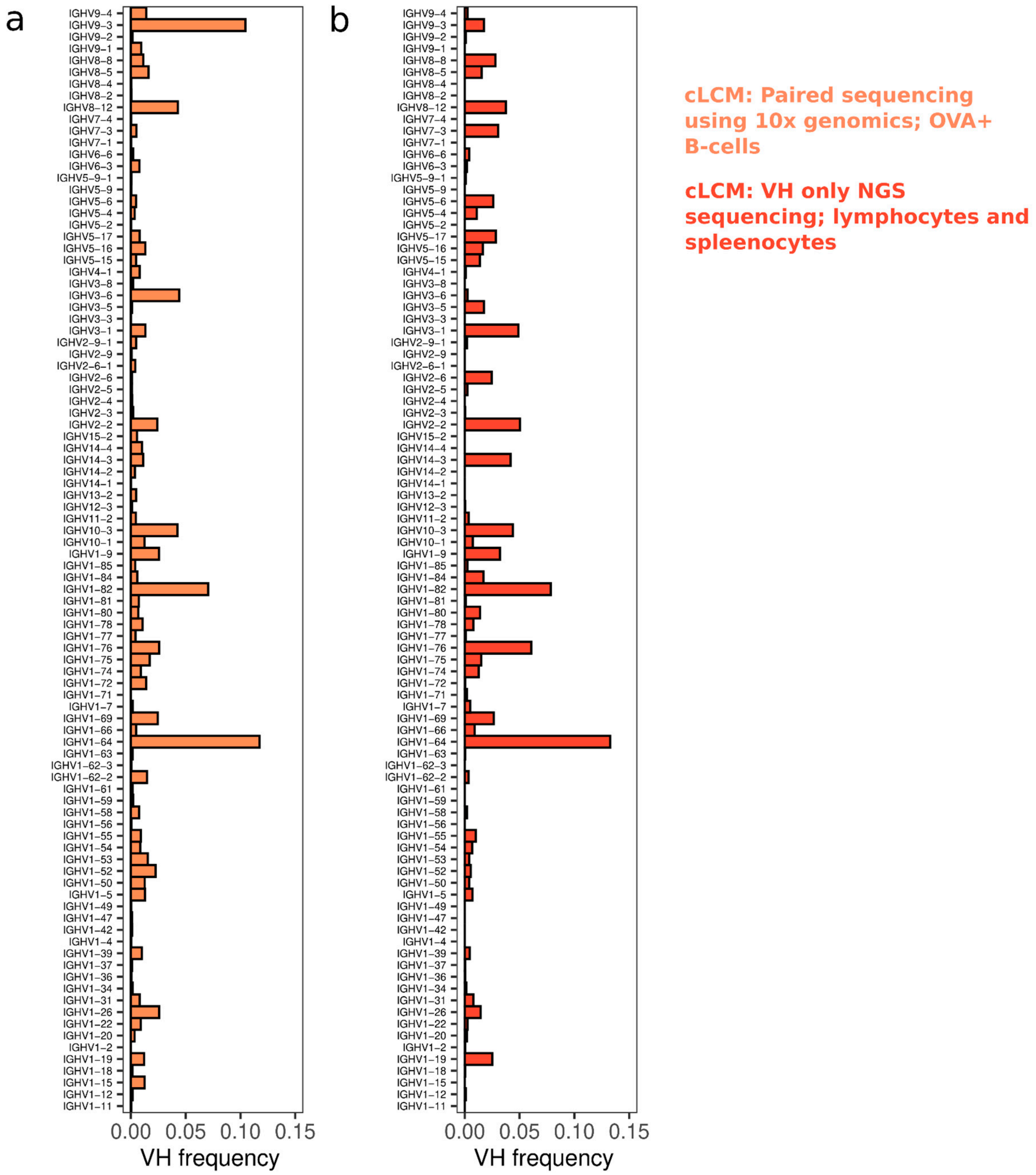

Supplementary Figure S8: Comparison of the IGHV gene usage in the antibody repertoire of the cLCM sequenced (a.) using 10x genomics paired seq (*n*=3859 sequences) and (b.) VH only repertoire sequencing (*n*=199461 sequences).

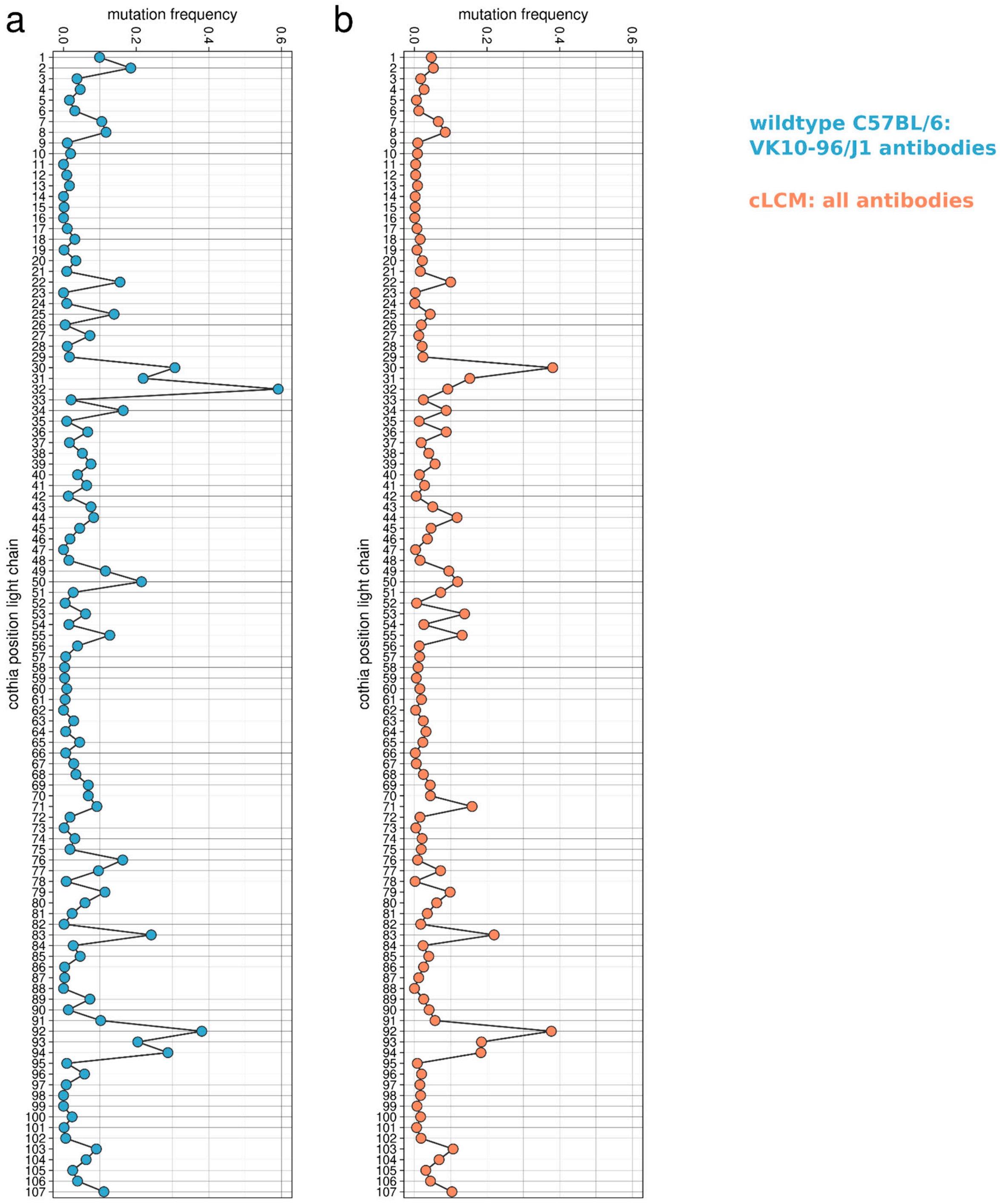

Supplementary Figure S9: Observed frequency of non-sense mutation from SHM resulting in amino acid changes for position 1 to 107 antibodies carrying IGKV10-96-J1 derived light chains in (a.) C57BL/6 (*n*=675 sequences) or (b.) cLCM (*n*=3420 sequences).
